# Artificial Intelligence in Glioblastoma—Transforming Diagnosis and Treatment

**DOI:** 10.1186/s41016-025-00399-2

**Published:** 2025-06-02

**Authors:** Alen Rončević, Nenad Koruga, Anamarija Soldo Koruga, Robert Rončević

**Affiliations:** 1 Department of Neurosurgery, University Hospital Center Osijek, 31000 Osijek, Croatia; 2 Faculty of Medicine, Josip Juraj Strossmayer University of Osijek, 31000 Osijek, Croatia; 3 Department of Neurology, University Hospital Center Osijek, 31000 Osijek, Croatia; 4 Department of Diagnostic and Interventional Radiology, University Hospital Center Osijek, 31000 Osijek, Croatia

**Keywords:** Artificial Intelligence, Chemotherapy, Glioblastoma, Immunotherapy, Neuro-oncology, Personalized therapy, Radiotherapy

## Abstract

Glioblastoma (GBM) is the most aggressive and common primary brain malignancy in adults, characterized by poor prognosis and treatment resistance. Despite advancements in treatment options, the median survival is roughly 15 months, underlining the need for novel and effective treatments. Artificial intelligence (AI) has emerged as a transformative technology in healthcare, offering outstanding capabilities in data analysis, pattern recognition, and helping in decision-making. This review explores the current and potential role of AI in GBM care, focusing on its applications in diagnosis, treatment planning, prognostication, and drug discovery. AI-based algorithms have demonstrated promising potential in enhancing diagnostics through imaging analysis, radiomics, and tumor segmentation. These technologies could enable non-invasive molecular profiling and early detection of GBM. In treatment planning, AI could improve approaches by optimizing surgical resection, radiotherapy regimen, and chemotherapy protocols. Furthermore, machine learning models can integrate multimodal data to develop personalized treatments. They can also enhance prognostication by predicting survival, recurrence, and treatment responses, helping clinicians to make more informed decisions. AI is also revolutionizing pharmacotherapy by identifying novel molecular targets and optimizing combination therapies. Despite notable progress, challenges persist. Limited data quality and quantity, algorithm interpretability, integration problems, and ethical considerations, remain significant challenges to clinical implementation. This review emphasizes the need for continued research and interdisciplinary collaboration to overcome many barriers and realize the transformative potential of AI in GBM care.

## Background

Glioblastoma (GBM) is the most aggressive and common primary malignancy of the brain [1]. It is characterized by the rapid progression despite first-line therapy and poor prognosis. Although some advancements in neurosurgical techniques, radiotherapy (RT), and chemotherapy (CRT) have been achieved, the median survival for patients with GBM remains poor [1]. This underlines the urgent need for innovative and personalized approaches to improve outcomes [2]. The complexity, intra-tumoral and inter-tumoral heterogeneity of GBM, coupled with other biological barriers such as the blood–brain barrier, further complicates its treatment.

In recent years, artificial intelligence (AI) has emerged as a transformative tool in preclinical studies and clinical practice, offering outstanding capabilities in large data analysis and pattern recognition, guiding decision-making in healthcare [3]. The rapid implementation of the digital tools, was in part influenced by the COVID-19 pandemic and the consequences it had on clinical practice [4–6]. By leveraging big data and advanced computational models, AI has demonstrated significant potential to revolutionize the field of neuro-oncology, i.e. the diagnosis, treatment, and monitoring of complex diseases, such as GBM [7, 8]. From improving on radiological imaging to guiding the path to personalized treatments, AI is reshaping the field of neuro-oncology [9].

This review explores the growing implementation of AI in GBM care, focusing on its implementation in diagnosis, treatment, patient stratification, prognostication, and therapeutic innovation. Potential roles of current AI models in GBM care are summarized in Table 1. Additionally, we discuss the challenges associated with AI in clinical practice and consider future directions for research and development in this rapidly changing area. By integrating AI into the care of GBM patients, there is a potential for overcoming current barriers and moving towards improved patient outcomes.

**Table 1:** Applications of AI tools in glioblastoma care

Stage	AI Application	AI Tools / Models	Clinical Utility
Diagnosis	Imaging classification	Deep learning CNNs, Radiomics [10]	Molecular profiling, tumor grading
	Tumor segmentation	U-Net [11], DeepMedic [12], nnU-Net [13]	Tumor volume estimation
Treatment Planning	Surgical navigation and planning	AI-assisted image systems [14]	Precise resection, reduced complications
	RT optimization	Reinforcement learning [15]	Lower toxicity, improved therapeutic response
	CRT response prediction	Multi-omics ML models [16]	Personalized drug regimens
Prediction	Survival prediction	Random Forests [17], XGBoost [18]	Patient counselling, risk stratification
	Recurrence risk estimation	Deep learning CNNs [19]	Timely intervention, risk stratification

*AI* artificial intelligence, *CNNs* convolutional neural networks, *CRT* chemotherapy, *ML* Machine model.

### AI in GBM diagnosis

The current framework for GBM diagnosis is dependent on the acquired tissue samples during the tumor resection [20]. However, even before the surgery is initiated, conventional radiological imaging, i.e. computed tomography (CT) and magnetic resonance imaging (MRI), can pre-operatively suggest a diagnosis of the GBM [21]. AI is enhancing the accuracy of GBM diagnosis based on pre-operative imaging. Advanced AI algorithms, such as those based on convolutional neural networks (CNNs) and deep learning, have demonstrated promising capabilities in analyzing neuroimaging data related to GBM [10]. Certain subtle features of the tumor can be overlooked because traditional radiological imaging interpretation relies on the radiologist's expertise. However, by spotting irregularities that radiologists might miss, these algorithms can improve the accuracy of diagnosis [22].

One of the main applications of AI in GBM diagnostics is radiomics. Radiomics is the extraction of numerous quantifiable data features from radiological images [23]. The extraction of those features produces datasets which can be utilized by AI to improve the care of GBM [24]. Radiomics-based AI models can differentiate between GBM and other brain lesions [25], predict tumor grade and prognosis [26], evaluate treatment response [27], and assess tumor subtypes non-invasively [28]. Furthermore, a bivariate meta-analysis performed by Di Salle et al. [29] showed that radiomics can achieve good performance in identifying isocitrate dehydrogenase (IDH) mutation status, a distinguished biomarker in GBM classification. However, aforementioned studies have some important caveats. As the authors have discussed, the quality of the studies and methodology is variable. Hence, the generalizability of these algorithms is unconfirmed. Finally, these algorithms are sometimes unable to outperform advanced imaging modalities. Another area where AI can help the clinicians is the segmentation, which is a process of distinguishing tumor tissue from the surrounding normal tissue [30]. The segmentation is a time-consuming process performed by expert radiologists so the implementation of AI could significantly improve this process. As presented by Zheng et al. [31], a deep learning model can achieve accurate segmentation of liver tumors. However, healthy brain and tumor tissues are more complex than the liver and translation to GBM segmentation is still out of reach. In fact, early attempts at segmentation of GBM failed due to the heterogeneity of the tumor tissue, but a method developed by Espa et al. [32] showed encouraging results. It would be useful to evaluate this method across multiple centers before implementation in clinical practice.

Furthermore, AI has the potential to facilitate early detection of GBM by quickly analyzing imaging data in asymptomatic individuals or those with subtle symptoms. sSozer et al. presented the case of a patient with non-specific headache who underwent MRI and was subsequently diagnosed with a glioma tumor [33]. By analyzing data from multiple imaging modalities, AI systems can generate comprehensive diagnostic insights, paving the way for timely and targeted interventions.

### AI in GBM treatment planning

As we have already discussed, there is an urgent need for personalized approaches to GBM treatment [2], and AI can help in choosing the most appropriate treatment plan, as well as the implementation of personalized therapeutic options. For example, CNNs can be used to accurately predict the possibility of sufficient tumor resection [34]. Furthermore, the implementation of intraoperative MRI with AI algorithms can maximize the extent of resection while preserving neurological function of the patient [14]. However, although AI has great potential in helping with intraoperative decision-making, some obstacles are still present and remain unsolved [35]. For example, studies often focus on AI tools to predict resectability, whereas neurosurgeons assess tumor location, size and other factors for treatment decisions. Furthermore, neurosurgeons present an optimistic bias regarding postoperative deficits which could influence AI algorithms [35].

The integration of AI into neurosurgical practice could transform the precision and safety of procedures [35]. In a study by Fabelo et al. [36], CNNs integrated with hyperspectral imaging provided accurate intraoperative distinction of healthy and tumor tissue, with a sensitivity and specificity of 88% and 100%, respectively. Furthermore, computer vision combined with ML tools could provide real-time guidance for neurosurgeons resulting in better outcomes [37]. Although this technology hasn’t been evaluated in GBM resection, it would be of great interest to neurosurgeons. Another area that is gaining more traction is surgical robotics [37]. The DaVinci surgical robot is among the most known in this area. However, due to numerous limitations, such as the lack of haptic feedback [38], it is currently not optimal for microneurosurgical approaches. Further technological improvements are needed before it is incorporated into standard GBM care. Neurosurgeons often use neuronavigation (NN) for GBM resection [2]. However, NN becomes unreliable as surgery progresses due to intraoperative brain deformation, i.e. brain shift [39]. This problem can be corrected by ML and deep learning-based approaches which can dynamically update NN data [40]. These models can improve the extent of resection and prevent injury to eloquent brain regions.

While most of these tools are still in experimental phases, their potential to improve surgeries, enhance precision, and reduce complications is considerable. Overcoming challenges is of vital importance before AI is routinely used in this part of GBM treatment.

Another avenue in GBM treatment planning that could be improved by the implementation of AI is RT. AI models could help in defining gross tumor volume and clinical target volume that could help in guiding RT protocols [41]. However, there is still a significant obstacle – most of these studies were performed on untreated tumors and not on GBM after resection, which is the common state when planning RT. Therefore these models will first have to show their efficacy in other circumstances before they are incorporated into clinical practice [42]. Furthermore, as the majority of GBM recurrences occur in proximity to the initial tumor [43, 44], this could be leveraged by AI algorithms and would help to modify the radiation dose to the target tissue [42]. Indeed, a computerized framework based on CNNs developed by Ebrahimi Zade et al. [45] showed promising results in designing personalized CRT and RT for GBM. Progress has been made regarding AI and RT planning, as reviewed by Sheng et al. [46]. Although the results are encouraging, they still need to be tested against bigger data to influence real-life treatment planning.

**Fig. 1:**
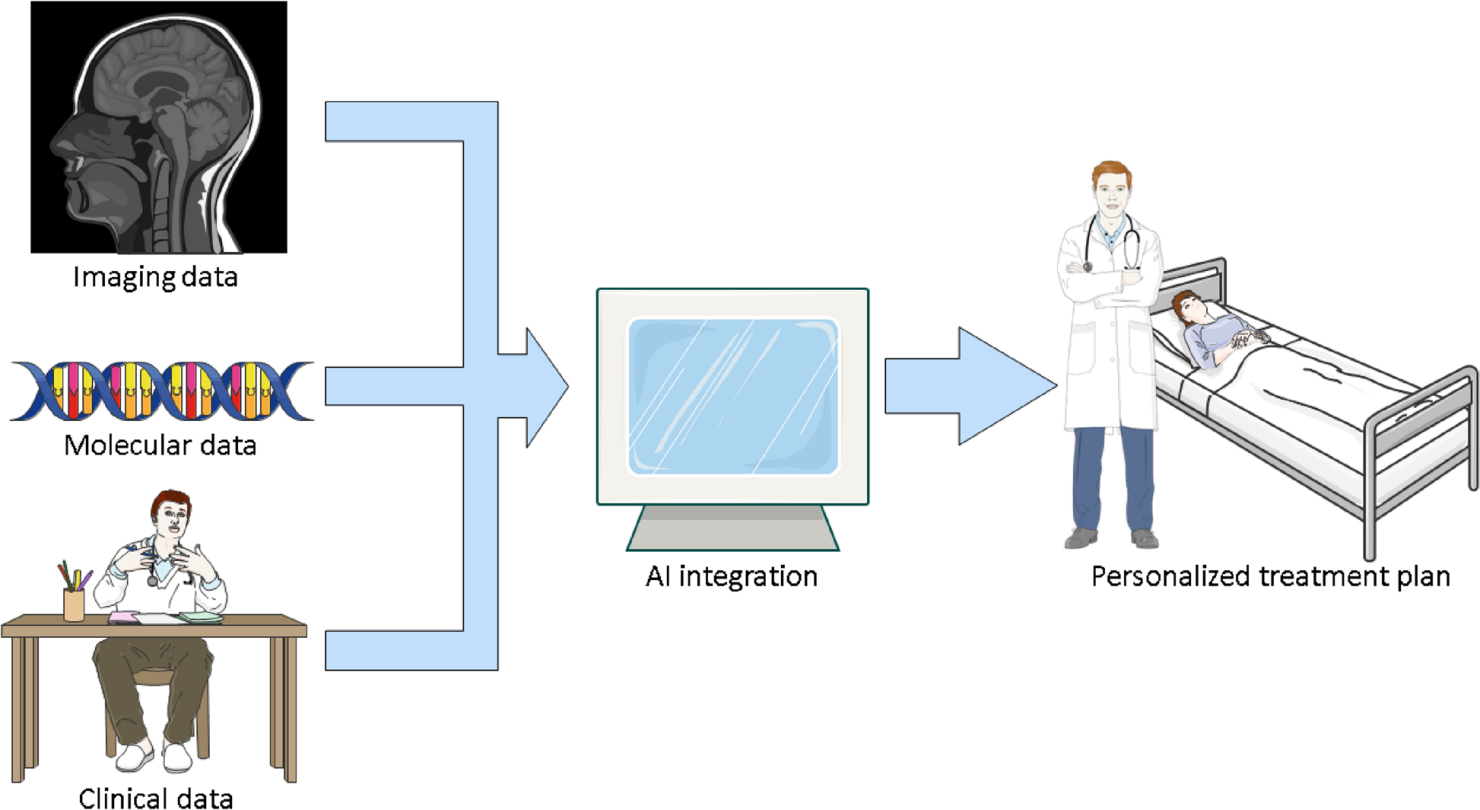
AI-driven personalized treatment planning. The figure shows the workflow of AI integration into clinical practice. Imaging, molecular, and clinical data are all evaluated and integrated by AI models which suggest the most optimal and patient-specific treatment plan

CRT protocols could also be personalized, and AI models can guide these changes. Temozolomide (TMZ) is an alkylating CRT agent routinely used as a part of Stupp regimen for treating GBM [47]. Interestingly, AI-based in silico models can be used to optimize the administration schedule of TMZ based on the tumor location and size, as well as patient characteristics [48, 49]. By utilizing machine learning (ML) and omics data researchers were able to accurately predict therapeutic response of low-grade gliomas to TMZ and achieve results which were superior to conventional biomarkers [50]. In addition, even large language models (LLMs) such as ChatGPT have presented impressive abilities regarding the adjuvant treatment plan for GBM patients [51]. On the other hand, the same study reported poor results of ChatGPT for identifying glioma subtypes. Currently, LLMs could be useful for patients by advising specialists visit, depending on the presenting symptoms [52]. Also, ChatGPT could be utilized for monitoring GBM patients and summarizing radiology reports for follow-ups [53]. Still, the problem of accountability and expanding knowledge capability of LLMs are unaddressed. It seems that these technologies are yet to be explored for specific neurosurgical applications. Advancing these and other algorithms and evaluating them on larger datasets and more heterogeneous patient populations could enable oncologists to select the most effective CRT agents and their dosages, reducing the likelihood of resistance and adverse effects. Moreover, AI models could integrate multimodal data, including imaging, molecular, and clinical information, to develop personalized treatment plans, which is illustrated in Fig. 1. These plans could be adjusted during the treatment regimen to changes of the tumor or the condition of a patient. By incorporating AI into treatment planning for GBM, clinicians can achieve a new level of personalization which was previously unattainable, paving the way for improved patient outcomes.

### AI in GBM prognostication

When considering potential treatment plans and choosing the most appropriate regimen, the most important metric is the patient outcome. The traditional approach of relying on clinical features did not prove to be consistent when predicting outcomes of these patients [54]. On the other hand, AI can significantly enhance prognostication and outcome prediction of GBM patients by relying on more relevant features. By utilizing several ML classifiers and preoperative MRI data, researchers were able to predict molecular characteristics of the tumor and the overall survival of studied patients [55]. As discussed by di Noia et al. [56], comprehensive features derived from preoperative MRI data can be effectively used for survival prediction of these patients. It should be noted that neuroimaging is also performed during the treatment of GBM, and imaging features obtained during follow-ups can also be used by AI models to evaluate the response to therapy, as well as to predict progression-free survival [57]. These insights could empower clinicians to make informed decisions tailored to specific individual needs.

One prominent application of AI is the development of survival prediction models. ML algorithms can analyze diverse datasets, including radiological, clinical, and therapeutic characteristics, and produce personalized survival curves which could also provide valuable cues for the optimal treatment plan [58]. Even more advanced algorithms, i.e. CNNs, displayed even more precise predictions and could serve as supplementary tools in treatment planning [59]. These models could identify patients who may or may not benefit from more aggressive or experimental therapies.

GBM is characterized by relatively quick recurrences [43]. Fortunately, predicting the recurrence patterns is another critical area where AI can have major influence. By evaluating radiomic features of contrast enhanced MRI, CNNs models were able to accurately predict local or distant recurrences of the tumor [60]. Identifying patients at high-risk of local recurrence is of utmost importance and ML models demonstrate high discriminative ability [61]. This enables timely intervention and may improve long-term outcomes. Additional problem concerns determining the difference of true tumor recurrence and treatment-related effects. Curiously, ML models based on multimodality MRI radiomics can even help with this task, supporting appropriate clinical decision making [62].

Stratifying patients diagnosed with GBM into separate groups is useful in order to develop more precise therapies, and this stratification usually relies on potential molecular biomarkers [63]. By combining clinical, molecular and imaging features, Ius et al. [63] were able to stratify patients into 5 groups with ML algorithm which could additionally enhance the survival predictions of GBM patients postoperatively. It would be beneficial to also predict treatment response based on tumor biology, which would ensure that patients are treated with therapies most likely to be effective. This personalized approach would not only enhance trial success rates but also minimize unnecessary exposure to ineffective treatments.

Furthermore, explainable AI models are gaining traction in oncology [64], providing insights into the factors driving predictions. This kind of model could improve attitudes of clinicians towards AI and facilitate the implementation of AI tools into routine practice of GBM care [65]. As these technologies continue to improve, along with the comfortability of clinicians of using those same technologies, there is a potential to redefine GBM prognostication, guiding precision medicine and improving patient care [66].

### AI in drug discovery and repurposing

Although current first-line treatment protocols have improved GBM patient outcomes, the overall survival and progression-free survival are still disappointing [1]. There is a growing need for finding novel and more effective treatment options. AI is transforming the landscape of drug discovery and repurposing by identifying potential therapeutic targets and optimizing existing drugs [67, 68]. The traditional drug discovery is a costly and lengthy process, and AI-based approaches could significantly reduce this timeframe [69]. Unfortunately, traditionally tested therapeutic agents for GBM have not been as successful [70]. Even though some changes to preclinical evaluation of potential therapeutics for GBM have been proposed [71], the implementation of AI algorithms in the process is still not as prevalent [72, 73]. As we have mentioned, the application of AI to drug repurposing would enhance this process [74]. By analyzing properties of a drug and molecular interactions with tumor cells, AI algorithms could suggest which existing drugs might be effective against GBM. As has been presented for COVID-19 [75], AI can optimize combination therapies by predicting synergistic effects between drugs. This could be particularly useful for GBM, where monotherapies often fail due to tumor heterogeneity and resistance [2, 76]. ML models can evaluate complex data to identify previously unrecognized pathways relevant for GBM growth and resistance mechanisms [77]. These insights could pave the way for the development of personalized therapies tailored to the unique molecular profile of GBM.

### Limitations and ethical considerations

The implementation of AI into routine GBM diagnosis and treatment is not without challenges and ethical dilemmas. One of the major barriers is limited quality and quantity of data [78]. Standardized frameworks and subsequentially high-quality datasets are essential for training AI models [79], yet GBM data is often limited due to various reasons [80]. There is a notable variability in published GBM datasets regarding imaging protocols, genetic sequencing, and clinical standards across different institutions. This can lead to inconsistent model performance across institutions and limit the generalizability of AI. Another problem is the absence of multicenter validation which hinders the translation of AI tools into clinical practice. Federated ML is a potential solution to data-related limitations. It enables multicenter collaboration without exchanging private patient data [81]. Collaboration across different institutions regarding research and care for GBM, as well as the development of standardized frameworks are crucial to overcoming these limitations. Another problem is the difficulty of interpreting AI algorithms which complicates their application in current practice [82]. Many AI models operate as "black boxes," generating predictions without explainable insights into their decision-making processes. In consequence, this lack of transparency hinders the widespread adoption of AI in clinical settings [83].

Algorithmic bias is another concern which results from unbalanced or non-representative training data. This is especially important for GBM which is not that common of a disease. This bias is further exacerbated if the studies are performed in a single institution. Lately, efforts have been made to develop explainable AI (XAI) models that are by nature more transparent, as these would be more easily accepted by healthcare workers [84, 85]. By uncovering decision-making, XAI could more easily conform to regulations and ethical oversight. However, further studies of XAI in GBM care are much needed. Still, effective integration of AI into clinical workflow poses logistical and technical challenges. To begin with, AI tools should be implemented into current systems without interruption of the workflow. This would require user-friendly interfaces, interoperability with electronic health records, and real-time processing capabilities.

Ethical considerations also play a pivotal role in the successful implementation of AI in GBM care. Preserving privacy of patients is a predominant concern, especially when dealing with sensitive health data [86]. Ensuring compliance with data protection regulations is essential to safeguard patient information and speed up the implementation of AI in healthcare [87]. Additionally, biases in datasets can lead to biased predictions across different patient populations, which requires continues external validation and re-calibration [88]. Furthermore, regulatory and legal frameworks for AI in healthcare are still evolving, adding another layer of complexity [89]. The lack of well-defined guidelines for the development, validation, and approval of AI tools in healthcare can delay their implementation. In the United States, the Food and Drug Administration (FDA) has introduced ‘Artificial Intelligence/Machine Learning (AI/ML)-Based Software as a Medical Device (SaMD) Action Plan’ which introduced regulatory framework for software, including AI-based tools. Furthermore, the European Union’s CE marking process evaluates the compliance of AI tools with current standards under the Medical Device Regulation. Regulatory trends are focused on explainability, data robustness, and surveillance to ensure reliability of AI tools. These regulations are crucial for the responsible integration of AI into GBM care and will shape the future innovations. Lastly, the resource requirements for AI systems may limit their accessibility, particularly in low-resource settings [90]. Ensuring that these technologies are accessible is crucial to prevent widening the gap between healthcare systems worldwide. Key limitations of integrating AI into standard GBM care are presented in Table 2. Addressing these challenges and ethical considerations is crucial to realize the full potential of AI in GBM care. By fostering transparency, collaboration, and inclusivity, the implementation of AI can be guided toward improved outcomes, ultimately transforming the landscape of neuro-oncology.

**Table 2:** Key challenges in implementing AI into GBM care

Challenge	Description	Proposed solutions
Data scarcity	Limited access to quality data	Institutional collaboration, open databases
Algorithm interpretability	Lack of transparency in decision-making process	Explainable AI models
Integration into workflow	Technical and logistical barriers	User-friendly interfaces, interoperability
Ethical concerns	Data privacy, algorithm bias	Robust validation, adherence to regulations

*AI* Artificial intelligence.

The implementation of AI into GBM care is still in early stage, with room for innovation and growth. There is a lot of potential to improve diagnostics, personalization of therapy, and clinical decision-making. Deep learning-based molecular subtyping provides non-invasive, image-based prediction of relevant molecular markers such as IDH mutation status and others [91]. These models could enable faster and accessible biomarker profiling, especially in resource-limited settings. As they further improve, there may be less dependence on invasive sampling. Reinforcement learning (RL) is another interesting method, which simulates treatment and responses [48]. This could be utilized to design patient specific RT and CRT for GBM patients. As a study by Ebrahimi Zade et al. [48] suggests, RL frameworks may perform better than traditional treatment which should be further investigated.

As these and other technologies evolve, collaboration will be indispensable for safety, transparency, and successful implementation of AI. Future studies should focus on multicenter cooperation, prospective validation, and open data, to realize the full potential of these innovations in GBM care.

## Conclusion

The integration of AI into GBM diagnosis, treatment planning and prediction marks a transformative shift in the management of this devastating disease. From improving diagnostic accuracy and treatment planning to advancing drug discovery and improving outcome prediction, AI-driven technologies could redefine the standards in neuro-oncology. While significant limitations remain, including data challenges, ethical concerns, and the requirement for undisruptive clinical application, advancements in AI research and continuous collaboration across disciplines and departments provide a path forward.

By addressing many of these challenges and promoting transparency, inclusivity, and innovation, AI can fulfil its promising potential in the fight against GBM. However, the goal remains: to improve patient outcomes, extend survival, and enhance the quality of life for patients with GBM. The future of improved GBM care lies at the intersection of novel and transformative technologies and improved clinical practice, offering hope in the face of one of the biggest challenges in modern medicine.

## Data Availability

Not applicable.

## Abbreviations

AI: Artificial intelligence
CNNs: Convolutional neural networks; CRT: Chemotherapy; CT: Computed tomography
GBM: Glioblastoma
IDH: Isocitrate dehydrogenase
LLM: Language learning model
ML: Machine learning; MRI: Magnetic resonance imaging
NN: Neuronavigation
RL: Reinforcement learning; RT: Radiotherapy
TMZ: Temozolomide
XAI: Explainable artificial intelligence
